# Presentation of chronic myeloid leukemia in basophilic blast crisis

**DOI:** 10.1002/ajh.27464

**Published:** 2024-08-23

**Authors:** Biswadip Hazarika, Barbara J. Bain

**Affiliations:** ^1^ Department of Haematology Batra Hospital and Medical Research Centre New Delhi India; ^2^ Centre for Haematology, Department of Immunology and Inflammation, St Mary's Hospital Campus of Imperial College Faculty of Medicine St Mary's Hospital London UK

A 65‐year‐old man presented with fatigue. His spleen was just palpable below the left costal margin. His blood count showed hyperleukocytosis (WBC 242 × 10^9^/L) with blast cells predominating, hemoglobin concentration (Hb) 64 g/L, and platelet count 22 × 10^9^/L. Strikingly, the majority of blast cells had cytoplasm packed with large basophilic granules; others had more scanty granules so that the typical chromatin pattern of a blast cell was clearly evident (image, Jenner−Giemsa stain ×100 objective). The manual differential count showed 74% blast cells, 6% myelocytes, 4% neutrophils, 2% lymphocytes, and 14% mature basophils. Flow cytometric immunophenotyping on the peripheral blood sample showed around 90% weakly CD45+ blast cells, which were positive for CD13, CD33, CD117 (weak), CD34, CD38, and CD7 and were negative for CD19, CD20, CD10, cytoplasmic(c) CD79a, CD14, CD64, cCD3, HLA‐DR, and cMPO. Reverse transcription polymerase chain reaction (RT‐PCR) on the peripheral blood sample showed a b3a2 *BCR::ABL1* (p210) transcript. The bone marrow karyotype was 46,XY,t(9;22)(q34;q11.2)[16]//47,XY,idem,+i(17)(q10) [4]. The differential diagnosis at this stage was acute myeloid leukemia (AML) or blast transformation of chronic myeloid leukemia (CML). With financial restraints, treatment was initiated with imatinib and hydroxycarbamide (hydroxyurea). One week later the acute phase of the disease had remitted with the features now being typical of chronic phase CML. The blood count now showed WBC 45.18 × 10^9^/L, Hb 83 g/L, neutrophils 74%, lymphocytes 3%, monocytes 4%, myelocytes 10%, and basophils (all mature) 9%. No blast cells were detected. RT‐PCR again showed a b3a2 *BCR::ABL1* (p210) transcript. Cytogenetic analysis showed persistence of t(9;22), but the isochromosome 17 was no longer present. The reversion to chronic phase disease with persistence of t(9;22) and *BCR::ABL1* confirmed a diagnosis of CML presenting in blast crisis rather than AML.

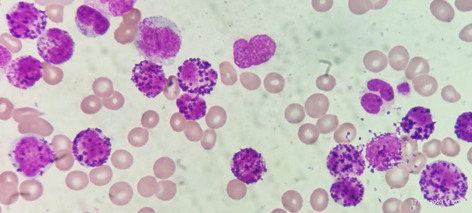

Basophilia with mature and dysplastic basophils is a common phenomenon in CML. However, a basophilic blast crisis is a very rare form of acute transformation. A review of 410 patients with disease in transformation revealed only two cases (0.5%) of basophilic blast crisis.^1^ The dominance of basophil blast cells with fewer or no other myeloblasts or lymphoblasts confirms the diagnosis. Clonal evolution is likely to be evident with additional chromosomal abnormalities, often including i(17q) or other abnormality of chromosome 17.^1^, ^2^, ^3^, ^4^ Diagnosis is straightforward when a patient is known to have CML but is more problematic if a patient presents already in blast crisis.

## CONFLICT OF INTEREST STATEMENT

The authors declare no conflict of interest.
